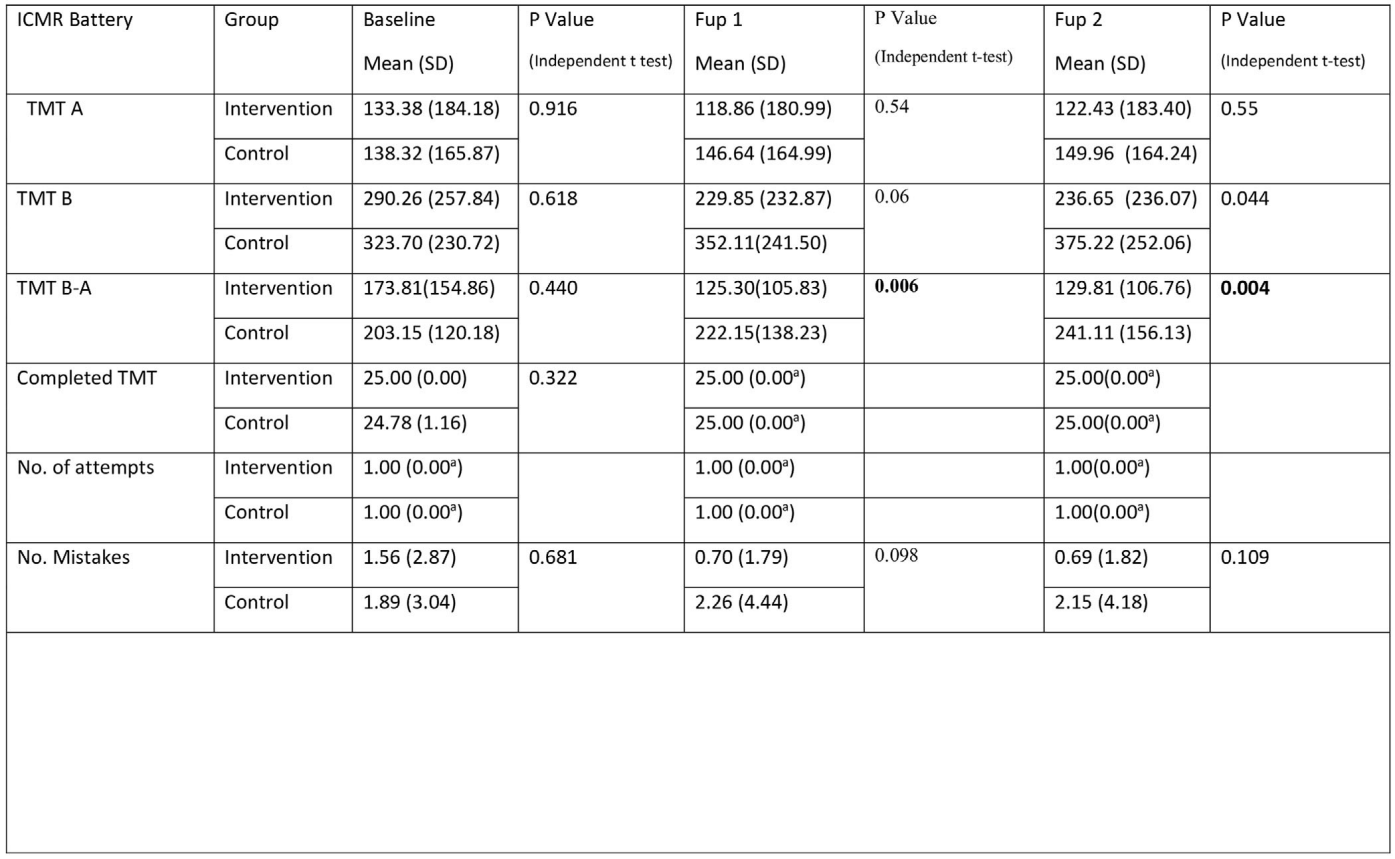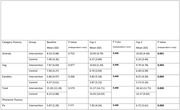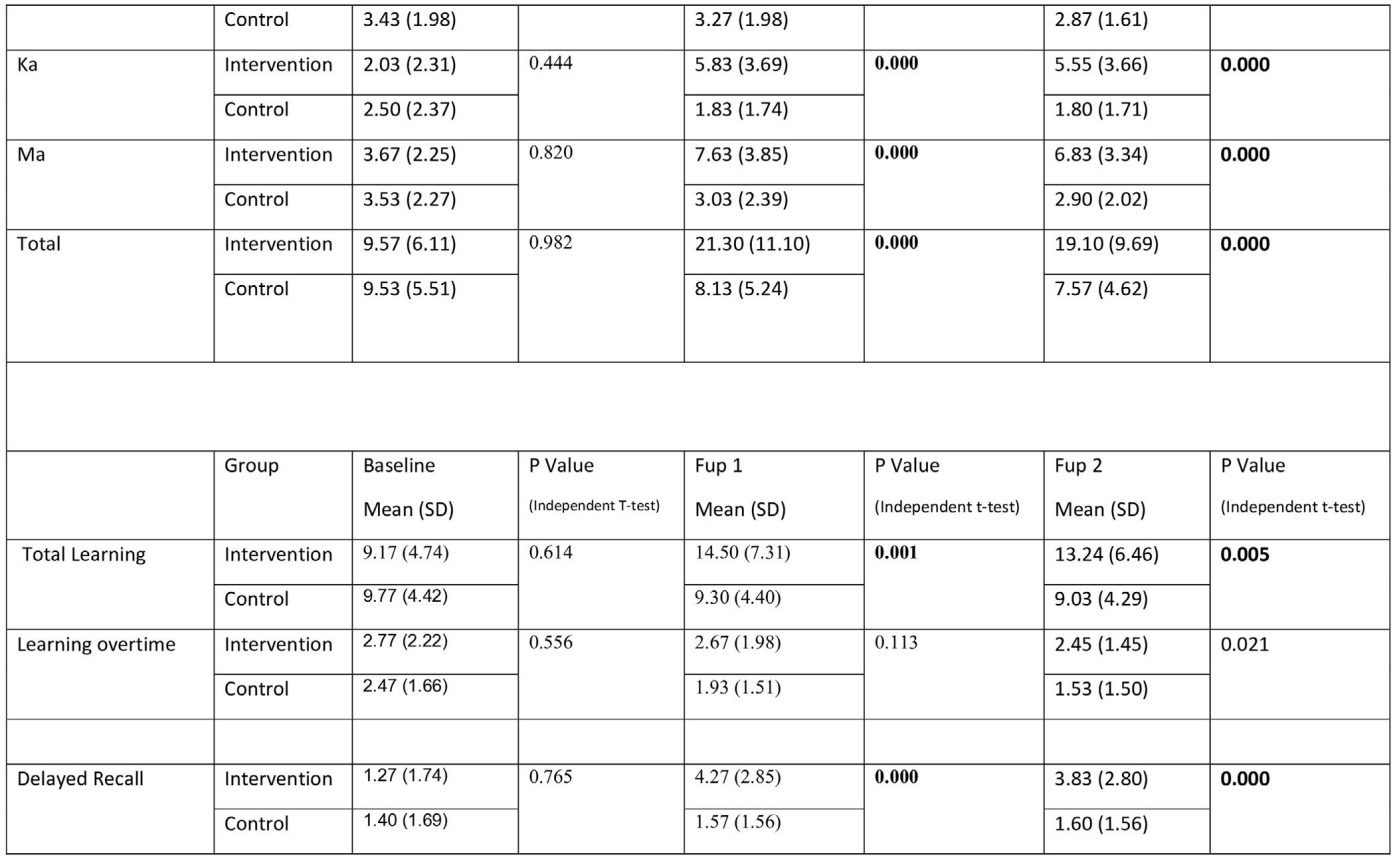# Cognitive Rehabilitation and Physical Activity for Early Dementia ‐ A Randomized Clinical Trial

**DOI:** 10.1002/alz.090354

**Published:** 2025-01-09

**Authors:** Mandeep, Dheeraj Khurana, Sucharita Ray, Priti Arun, Jyoti Mishra, Manreet Kaur, Ashok Kumar

**Affiliations:** ^1^ Post Graduate Institute of Medical Education and Research, Chandigarh, Chandigarh India; ^2^ Government Medical College & Hospital, Chandigarh, Chandigarh India

## Abstract

**Background‐:**

An estimated 8.8 million Indians older than 60 years have dementia. The increase in number of people living with dementia is causing difficultly to meet the need for care and support, particularly people with early‐stage dementia. Pharmacological treatments for dementia might not be effective or tolerated by all due to its possible side effects. Cognitive rehabilitation and physical activity may help preventing or slowing progression of dementia

**Objective‐:**

To find efficacy of combined cognitive and physical intervention in improving the cognition in people with Mild Cognitive Impairment (MCI) and Early Dementia.

**Methods‐:**

A randomised clinical trial was conducted among 60 patients with MCI and early dementia. The Intervention arm received Cognitive Rehabilitation and Physical Activity intervention along with Standard Care (n = 30) and Control arm received standard care (n = 30). Follow‐ up assessments were conducted at 1 month and 3 months. The primary outcome measure were attention, memory and executive functions. ADLs and IQCODE were the secondary outcomes. Descriptive statistics was used for statistical analysis using SPSS‐22 software.

**Results:**

Among 60 randomized participants (mean age, 62.5 years; 70% male) 22 (73%) of intervention arm completed the cognitive retraining worksheets. At 1 month and 3 months, Intervention arm showed significant improvement in TMTB‐A, Category fluency, Phonemic fluency, Total learning and Spatial recall (mean difference ‐98.455, P = 0.00; 16.40, P = 0.00; 15.80, P = 0.00; 6.59, P = 0.00; 2.79, P = 0.002 at Fup 1 and ‐150.36, P = 0.00; 16.49 P = 0.00; 14.7, P = 0.00; 6.86, P = 0.00; 3.26, P = 0.002 at Fup 2). No statistically significant difference as fount in IADL and OQCODE.

**Conclusions‐:**

In patients with MCI and early Dementia, Combined Cognitive and Physical Intervention in addition to Standard of Care may show improvement Attention, Memory and Executive Functioning.